# The Effect and Evaluation of the Third Military Medical University Fluid Resuscitation Formula

**DOI:** 10.1155/2022/8984696

**Published:** 2022-06-20

**Authors:** Jia Luo, Peng Zhang, Ying-Hong Gan, Ning Li, Li-Li Yuan, Gao-Xing Luo, Fei Xiang

**Affiliations:** Institute of Burn Research, State Key Laboratory of Trauma, Burns and Combined Injury, Southwest Hospital, Third Military Medical University (Army Medical University), Chongqing, China

## Abstract

**Background:**

The clinical efficacy of the third Military Medical University formula (TMMU formula) for fluid resuscitation stage was evaluated to improve the treatment level of adult patients with extensive burns during the shock stage.

**Methods:**

Retrospective analysis of the data of 55 patients undergoing fluid resuscitation according to the TMMU formula within six hours after burn injury. The following indicators were collected: (1) demographic and injury information; (2) fluid resuscitation information; (3) efficiency information, including cardiovascular function, liver function, renal function, coagulation function evaluation indicators, blood concentration, and average urine output index.

**Results:**

(1) In the first and second 24 hours after injury, the median fluid rehydration coefficient was 1.68 ml/kg·(%) TBSA and 1.15 ml/kg·(%) TBSA, the median ratio of crystal to colloid was 2.24 and 1.67, and the median urine output index was 0.75 ml/kg·h and 1.05 ml/kg·h, respectively. (2) The actual fluid volume during patient resuscitation is higher than the formula calculated volume, and this difference is more obvious in patients with burn area ≥80%. (3) In the second 24 hours, the value of the actual total fluid volume minus the formula total volume in the group with crystal to colloid ratio ≤2 was significantly lower than that in the ratio >2 group. (4) At 24 and 48 hours after injury, the cardiovascular function, liver function, renal function, and coagulation function were better than those before fluid resuscitation.

**Conclusions:**

Early application of the TMMU formula for fluid resuscitation in adult patients with extensive burns is safe and effective, but the actual input volume often exceeds the volume calculated by the formula, especially in the second 24 hours after burn injury and in patients with larger burn areas. Increasing the colloid input volume can help reduce the total amount of fluid used for resuscitation.

## 1. Introduction

Burn shock is the main early complication of severe large-area burns. Active and effective shock prevention and treatment is essential to the successful treatment of patients with large-area burns [1]. Fluid resuscitation is currently the main method used to treat burn shock. Many treatment programs have been proposed worldwide for fluid resuscitation after burn injury, and these treatments are mainly divided into two types: crystalloid fluid supplementation and mixed crystal and colloidal fluid supplementation. The former is represented by the Parkland formula [2], and the latter is represented by the Brook formula and the third Military medical university (TMMU) formula [3, 4].

The Parkland formula is widely used in Western countries, and the TMMU formula is widely used in China. All formulas mentioned previously use burn area and body weight to predict the amount of fluid replacement and do not consider the impact of burn depth and other aspects [5]. In clinical practice, it has been found that patients with the same burn areas but different burn depths have substantial differences in terms of the amount of fluid needed per unit of body weight. With deeper burn depth and larger area, the actual input volume may be higher than the input volume predicted by the formula. Excessive fluid input allows patients with severe burns to temporarily pass the shock period and can cause organ-specific edema, abdominal compartment syndrome, multiple organ dysfunction syndrome, and other complications, which increase the difficulty of treatment and reduces the treatment success rate [6–8]. However, although the present formulas have some limitations, they are still the basis of fluid resuscitation in treating burn shock. Therefore, scientific and accurate treatment formulas to support fluid resuscitation are beneficial for helping burn patients smoothly pass the shock period. The purpose of this study was to review the clinical case data of adult patients with large-area burns admitted to the Burn Research Institute of Southwest Hospital of Army Military Medical University from January 2015 to December 2019, analyze the amount of liquid used for burn patients to prevent early shock and the treatment effect, and evaluate the clinical efficacy of the TMMU formula. This study provides some experience for the better implementation of formulas to guide liquid resuscitation treatment of burn shock and improve the success rate of the treatment.

## 2. Materials and Methods

### 2.1. Patients

**Figure 1:**
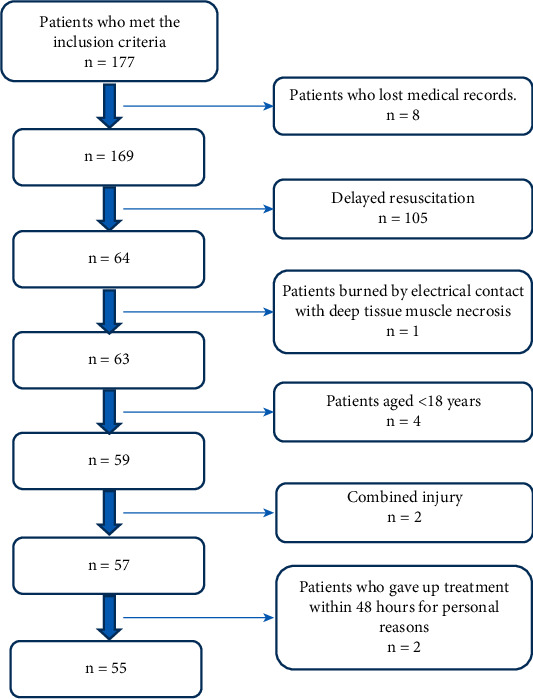
Inclusion and exclusion criteria from 177 patients admitted between January 2015 to December 2019.

This study included the clinical data of early adult patients with extensive burns who were admitted to the author's hospital from January 2015 to December 2019 (Figure 1).

*(1) Inclusion Criteria*. The inclusion criteria were as follows: patients with second- or third-degree burn area ≥50% total body surface area (TBSA).

*(2) Exclusion Criteria*. The exclusion criteria were as follows:

1. combined injury (patients with a combination of skin avulsion, fracture, intracranial hemorrhage, abdominal organ hemorrhage, and other compound injuries),
2. patients aged <18 years,
3. delayed resuscitation (patients admitted to the authors' department to receive fluid resuscitation outside 6 hours after injury),
4. patients burned by electrical contact with deep tissue muscle necrosis,
5. patients who gave up treatment within 48 hours for personal reasons,
6. patients who lost medical records.

This study followed the principles outlined in the Declaration of Helsinki and was approved by the Ethics Committee of Southwest Hospital, Third Military Medical University (Chongqing, China) (approval number: KY2020185). This study was retrospective, and patients' informed consent was exempted.

### 2.2. Methods

#### 2.2.1. Treatment

1. *Liquid Recovery.* After admission, all patients underwent fluid resuscitation in accordance with the principle of the TMMU formula. In the first 24 hours after injury, the amount of fluid was II° and III° burn area × weight (kg) × 1.5 ml ＋ water 2000 ml, the ratio of crystal to colloid fluid (RCC) was 2 : 1, half of the total fluid was injected in the first eight hours after injury, and the remaining fluid was injected in the last 16 hours. In the second 24 hours after injury, the crystal and colloid fluid amounts were half of those in the first 24 hours, and the water content remained unchanged. The crystal fluids were mainly Ringer lactate solution and 5% sodium bicarbonate solution, the colloid fluid was the same type of plasma (or 20% human blood albumin), and the water fluid was 5% glucose solution.
2. *Liquid Volume Adjustment.* The patient's heart rate, respiration, blood pressure, and peripheral blood oxygen saturation were monitored, the patient's hourly intake and outflow were recorded, the rate and volume of fluid rehydration were adjusted according to the patient's hourly changes in urine volume, and the urine volume was maintained at 0.5–1 mL/kg/h.

#### 2.2.2. Data Collection

1. Demographic and injury information, including sex, age, weight, injury factors, time to admission after burn, total burn area (TBA), and third-degree burn area, was collected.
2. Fluid resuscitation information, including the actual input volume and formula predicted input volume of crystals, colloid, water, and total fluids during the first and second 24 hours after burn injury, was collected. The fluid rehydration coefficient (FRC) and RCC were calculated for the first and second 24 hours after injury (FRC = (total fluid volume−2000)/(weight × TBA)).
3. Efficiency information, including heart rate (HR), hemoglobin (HB), hematocrit (HCT), mean arterial pressure (MAP), plasma osmotic pressure (POP), serum creatinine, blood urea nitrogen (BUN), lactic acid, alanine aminotransferase (ALT), aspartate aminotransferase (AST), and albumin and activated partial thromboplastin time (APTT) at the time of admission and at 24 hours and 48 hours after burn injury, was collected. The average urine output index (UI) was calculated for the first and second 24 hours after injury (UI = total urine output/(body weight × 24)).

### 2.3. Statistical Analysis

All data were analyzed using SPSS 24.0 software (SPSS, Inc., Chicago, IL). Due to the abnormal distribution, the results were reported as medians and interquartile ranges (IQRs), and nonparametric tests were employed. Paired data were compared by Wilcoxon signed rank test. Unpaired data were compared by the Mann–Whitney *U* test. *P* < 0.05 was considered statistically significant.

## 3. Results

### 3.1. Demographic and Injury Characteristics

A total of 55 patients with early extensive burns were included in this study. The patients included 46 males (83.6%) and 9 females (16.4%), with a ratio of 5.1 : 1.0. There were 49 patients (89.1%) with third-degree burns, and the second-degree burn area ranged from 0 to 95% TBSA, with the median being 27.0 (12.0, 50.0) TBSA. The duration of admission ranged from 107 to 358 minutes, with the median being 242 (204.0, 301.0) minutes. The patients' ages ranged from 21 to 75 years, with the median being 45.0 (29.0, 50.0) years. The TBA ranged from 51% to 95% TBSA, with the median being 72.0 (56.0, 82.0) TBSA. The weight ranged from 42.5 to 85.0 kg, with the median being 63.0 (55.0, 70.0) kg. The causes of injury were flames in 44 patients (80.0%), scalds in seven patients (12.7%), arc burns in two patients, and chemical burns in two patients (3.6%). During antishock therapy, none of the 55 patients received vasoactive drugs, and none developed complications such as heart failure, abdominal compartment syndrome, and MODS.

### 3.2. Fluid Intake Shock

In the first 24 hours after injury, the median FRC was 1.68 ml/kg (%) TBSA, and the median RCC was 2.24. In the second 24 hours after injury, the median FRC was 1.15 ml/kg (%) TBSA, and the median RCC was 1.67 (Table 1). In the first 24 hours after injury, the actual crystal water and total fluid volumes were all significantly greater than the formula-predicted volumes (*P* < 0.05), while the actual colloid fluid volume was not significantly different from the formula-predicted volume (*P* > 0.05). In the second 24 hours after injury, the actual crystal, colloid, water, and total fluid volumes were all significantly greater than the formula-predicted volumes (*P* < 0.05) (Table 2).

### 3.3. Effect of Burn Area on Fluid Volume

Patients were divided into a 50% to 79% group and *a* ≥ 80% group according to the burn area. In the first 24 hours after injury, the actual amount of total fluid volume in both groups was greater than the formula-predicted volume, and the actual total fluid volume in the ≥80% group was significantly greater than the formula-predicted volume (*P* < 0.05). However, there was no significant difference between the actual crystal and colloid volume and the formula-predicted volume, as well as the actual amount of total fluid volume and the formula-predicted volume in the 50% to 79% group (Table 3). In the second 24 hours after injury, the actual colloid, crystal, and total fluid volumes in the two groups were significantly greater than the formula-predicted volumes, and the actual water volume in the ≥80% group was significantly greater than the formula-predicted volume (*P* < 0.05) (Table 4).

### 3.4. Influence of Different Crystal-to-Colloid Ratios on the Amount of Liquid Rehydration

The RCC in the TMMU formula is 2. The patients were divided into two groups: RCC ≤ 2 group and RCC > 2 group. The results showed that, in the first 24 hours after injury, there was no statistical significance between the actual total fluid volume and the formula total fluid volume in the two groups. However, in the second 24 hours after injury, the difference value between the actual total fluid volume and the formula predicted volume in the RCC ≤ 2 group was significantly less than that in the RCC > 2 group; that is, more colloid input could reduce the total fluid volume in the second 24 hours after injury (Table 5).

### 3.5. Liquid Resuscitation Effect

All patients underwent fluid resuscitation according to the formula and passed through the burn shock period smoothly. In the first and second 24 hours after injury, the median UI was 0.75 ml/kg/h and 1.05 ml/kg/h, respectively. Further analysis of the three time points, namely, at admission, 24 hours, and 48 hours after injury, showed that the patients' HB and HCT levels at 24 hours and 48 hours after injury were significantly lower than those at admission, and the blood concentration was significantly improved, and the difference was statistically significant (*P* < 0.05). The patients' serum creatinine, BUN, lactic acid, ALT, and AST levels at 24 hours and 48 hours after injury were significantly lower than those at admission, and the liver and kidney function was significantly improved, and the difference was statistically significant (*P* < 0.05). At these three time points, the HR, MAP, and POP were all within the normal range. At 24 hours and 48 hours after injury, the APTT was not significantly different compared with those before rehydration (*P* > 0.05). At 24 hours and 48 hours after injury, the albumin was significantly lower than that at admission, and the difference was statistically significant (*P* < 0.05) (Tables 6 and 7).

## 4. Discussion

Large-area severe burns are difficult to treat and are a focus of burn treatment. In the early stage, shock is caused by increased vascular permeability and extravasation of fluid [9, 10]. The treatment of burn shock depends on prompt and effective fluid resuscitation. By definition, prompt means that the start of fluid rehydration treatment should occur as early as possible after injury, and effective means that the fluid resuscitation treatment should enable a patient to smoothly pass through the shock period and prevent various organ-related complications [3]. There are many formulas for fluid recovery in China; although these formulas differ, the core points are the alternating replenishment of crystals, colloids, and water. The TMMU formula has a good effect on fluid resuscitation in patients with burn shock [11], but the effect of this formula on early fluid resuscitation (≤6 hours after injury) in patients with large-area burns (≥50% TBSA) has not been reported recently. In this study, the TMMU formula was used as the standard. The results showed that, for patients with large-area burns with nondelayed resuscitation (fluid resuscitation began ≤6 hours after injury) [12], the urine volume of patients could be well maintained within 24 hours and 48 hours after injury. Because the patients included in this study were promptly hospitalized after injury, although their blood was clearly concentrated, they were generally in the compensatory stage of shock without obvious signs of shock, such as decreased blood pressure and increased heart rate, and the liver and kidney function was significantly improved. At 24 hours and 48 hours after injury, the albumin was significantly lower than that at admission, and it was considered caused by albumin dilution after increased circulating fluid. The previously mentioned results indicate that the TMMU formula is prompt, effective, and safe for fluid resuscitation in patients with large-area burns, and this approach can not only improve burn shock but can also prevent pulmonary edema and damage to the function of other organs [13].

The formula for fluid replacement is of guiding significance to the fluid resuscitation of burn patients, but it cannot be mechanically replicated without various in the actual treatment process. In the current rehydration formula, patients' burn area and weight are the decisive factors of rehydration volume. In addition, factors such as burn depth and inhalation injury also play an important role in the loss of fluid in patients with large-area burns. Burn scholars have conducted a number of studies and proposed a number of new schemes for fluid resuscitation [14, 15]. However, the newly proposed formulas are either too complex to adopt or have poor clinical application, so the original formula is still widely used. The formula for fluid replacement should not be mechanically applied in clinical practice. The ultimate goal of fluid replacement for burn shock is to provide the amount of fluid that is needed. In this study, the first and second 24 hours FRC, the actual amount volume, and the formula amount volume were compared in detail in patients with different burn areas. The results indicated that the actual volume within 48 hours after injury is greater than the formula-predicted volume, especially in the second 24 hours after injury, and this growing trend may be positively correlated with the size of the patient's burn area. That is, the larger the burn area is, the more fluid the patient actually needs, and the greater the range that exceeds the formula amount. Therefore, in the process of fluid resuscitation for patients with large-area burns, it is necessary to further refine the formula and individually modify the actual fluid rehydration volume based on the burned area and patient test indices to achieve perfect shock resuscitation.

There has always been a controversy about the components of the rehydration formula, which mainly include the crystal resuscitation scheme represented by the Parkland formula and the crystal colloid scheme, in which crystals and colloids are alternately supplemented. The Parkland formula is widely used in Europe and the United States, while the approach in China basically involves a crystal colloid scheme. As mentioned earlier, the actual volume delivered in the shock phase is generally greater than the formula-predicted volume, especially for burn patients who are administered only crystal fluid for resuscitation. The more severe the burn is, the greater the amount of fluid infusion is required. In the crystal scheme, excessive crystalloid input is prone to fluid creep, resulting in pulmonary edema, abdominal compartment syndrome, and other complications [1, 16]. In the crystal colloid scheme, the actual volume input is also larger than the formula-predicted volume, but the increase is not extreme [17]. Meanwhile, patients in this study were divided into RCC ≤ 2 group and RCC > 2 group. The results indicated that more colloids can significantly reduce the actual fluid rehydration amount, but there is no significant difference in this decreasing trend in the first 24 hours after injury. In addition to the insufficient sample size of this study, this result may also be due to the long time required for clinical blood matching and other processes in the early stage after burn injury, resulting in a limited time for actual colloid supplementation in the first 24 hours. The colloid supplementation is relatively insufficient. Recently, some researchers have conducted a series of discussions on the application of colloid-based scheme to avoid the previously mentioned complications due to excessive crystalloid supplementation [18, 19]. The currently used colloids include natural colloids (plasma and human albumin) and artificial colloids (dextran, hetastarch, and gelatin). Plasma is the preferred source of colloids at present, but patients with large-area burns often require a large amount of plasma for fluid resuscitation. It is difficult to provide such a large amount in clinical practice in some conditions. The appearance of artificial colloid is of great significance for relieving the lack of plasma. Hetastarch and dextran were proved to be used with caution or in small amounts during fluid resuscitation in burn shock because of their toxic and side effects [20–24]. Gelatin (e.g., Haemaccel and Gelofusine) is often used in burn shock resuscitation, but it is seldom reported that gelatin is used in large amount or completely instead of plasma in the process. Considering the advantages of colloids in fluid resuscitation of severe burns and the lack of natural colloids, it is necessary to make a prospective study on the use of artificial colloids with large doses in this field.

In this study, we confirm that early application of the TMMU formula in adult patients with severe large-area burns is safe and effective. However, the actual fluid rehydration volume often exceeds the formula-predicted volume in clinical practice, especially in the second 24 hours after injury and in patients with larger burn areas; these results indicate that increasing the colloid input is beneficial to reducing the total amount of fluid rehydration administered. Of course, this study is a retrospective analysis, and its conclusions still need to be further verified by large-sample, multicenter clinical studies.

## Figures and Tables

**Table 1:** FRC, RCC, and UI in shock stage.

	The first 24 hours	The second 24 hours
FRC	1.68 (1.23, 2.09)	1.15 (0.87, 1.42)
RCC (%)	2.24 (1.73, 3.05)	1.67 (1.24, 2.33)
UI (ml/kg/h)	0.75 (0.54, 1.05)	1.05 (0.73, 1.73)

FRC: fluid rehydration coefficient; RCC: ratio of crystal to colloid; UI: urine output index.

**Table 2:** The difference in rehydration volume in the shock stage.

Group		No.	Formula amount (ml)	Actual amount (ml)	W	*P*
The first 24 hours	Total	55	8804 (7250, 10400)	8901 (7082, 11121)	−2.287	0.022
	Colloid	55	2268 (1750, 2800)	2220 (1480, 2600)	−0.531	0.595
	Crystal	55	4536 (3500, 5600)	4600 (3545, 6065)	−2.036	0.042
	Water	55	2000.00	2210 (2000, 3065)	−3.836	<0.001
The second 24 hours	Total	55	5402 (4625, 6200)	7020 (5460, 8570)	−5.739	<0.001
	Colloid	55	1134 (875, 1400)	1710 (1350, 2290)	−5.756	<0.001
	Crystal	55	2268 (1750, 2800)	2821 (2236, 3708)	−4.793	<0.001
	Water	55	2000.00	2041 (1750, 3020)	−2.552	0.011

No.: number. All data were compared by Wilcoxon signed rank test.

**Table 3:** The difference in fluid rehydration in patients with different burn areas during the first 24 hours after injury.

Group		No.	Formula amount (ml)	Actual amount (ml)	W	*P*
50% to 79% group	Total	36	7775 (6761, 8812)	8407 (6629, 9313)	−1.304	0.192
	Colloid	36	1925 (1587, 2271)	1933 (1258, 2396)	−0.450	0.652
	Crystal	36	3850 (3174, 4541)	4163 (3351, 5457)	−1.445	0.148
	Water	36	2000.00	2175 (1936, 2438)	−2.422	0.015
≥80% group	Total	19	10400 (9503, 11113)	11111 (8901, 15572)	−1.972	0.049
	Colloid	19	2800 (2501, 3038)	2548 (2080, 3560)	−0.240	0.811
	Crystal	19	5600 (5002, 6075)	6065 (4851, 7550)	−1.368	0.171
	Water	19	2000.00	2700 (2000, 4650)	−3.051	0.002

No.: number. All data were compared by Wilcoxon signed rank test.

**Table 4:** The difference in fluid rehydration in patients with different burn areas during the second 24 hours after injury.

Group		No.	Formula amount (ml)	Actual amount (ml)	W	*P*
50% to 79% group	Total	36	4888 (4381, 5406)	6350 (5361, 8102)	−4.572	<0.001
	Colloid	36	963 (794, 1135)	1485 (1215, 1960)	−4.368	<0.001
	Crystal	36	1925 (1587, 2271)	2590 (2135, 3386)	−4.132	<0.001
	Water	36	2000.00	2000 (1750, 2925)	−1.353	0.176
≥80% group	Total	19	6200 (5752, 6556)	8002 (7020, 10199)	−3.501	<0.001
	Colloid	19	1400 (1251, 1519)	2190 (1710, 2420)	−3.783	<0.001
	Crystal	19	2800 (2501, 3038)	3335 (2700, 4975)	−2.455	0.014
	Water	19	2000.00	2400 (1700, 3020)	−2.288	0.022

No.: number. All data were compared by Wilcoxon signed rank test.

**Table 5:** Influence of different RCCs on the volume of fluid rehydration.

Group		RCC ≤ 2 group	RCC > 2 group	*Z*	*P*
The difference between actual and formula total fluid volume (ml)	No.	21	34	−0.450	0.652
	The first 24 hours	405 (−1350,2581)	943 (−813,2579)		
	No.	34	21	−2.235	0.025
	The second 24 hours	1062 (435,2570)	2781 (1089,3763)		

No.: number. RCC: ratio of crystal to colloid. All data were compared by the Mann–Whitney *U* test.

**Table 6:** Liquid resuscitation effect between admission and 24 hours after injury.

Group	No.	Admission	24 hours after injury	W	*P*
Hb (g/L)	55	183.0 (163.0, 199.0)	152.0 (138.0, 172.0)	−5.835	<0.001
HCT (%)	55	53.2 (47.4, 59.8)	43.9 (40.1, 51.9)	−5.595	<0.001
MAP (mmHg)	55	95.0 (84.7, 102.0)	95.7 (91.0, 109.3)	−1.546	0.122
HR (times/min)	55	107.0 (89.0, 125.0)	101.0 (86.0, 116.0)	−2.661	0.008
POP (mmol/L)	50	306.2 (297.7, 315.0)	304.1 (291.2, 310.6)	−3.519	<0.001
Lactic acid (mmol/L)	30	4.2 (3.1, 5.9)	2.5 (1.9, 3.8)	−3.426	0.001
BUN (mmol/L)	51	6.9 (6.1, 8.5)	6.2 (4.3, 8.1)	−1.533	0.125
Serum creatinine (mg)	51	79.8 (69.6, 101.2)	68.0 (57.6, 89.0)	−2.118	0.034
Albumin (g/L)	53	33.4 (29.1, 38.4)	26.9 (23.5, 30.1)	−5.294	<0.001
ALT (U/L)	52	34.0 (24.2, 46.8)	29.9 (20.5, 39.5)	−2.668	0.008
AST (U/L)	51	68.1 (56.2, 110.4)	40.2 (31.0, 55.5)	−5.212	<0.001
APTT (s)	38	32.6 (24.6, 59.1)	44.5 (33.5, 64.2)	−0.181	0.856

No.: number. HB: hemoglobin; HCT: hematocrit; MAP: mean arterial pressure; HR: heart rate; POP: plasma osmotic pressure; BUN: blood urea nitrogen; ALT: alanine aminotransferase; AST: aspartate aminotransferase; APTT: albumin and activated partial thromboplastin time. All data were compared by Wilcoxon signed rank test.

**Table 7:** Liquid resuscitation effect between admission and 48 hours after injury.

Group	No.	Admission	48 hours after injury	W	*P*
Hb (g/L)	55	183.0 (163.0, 199.0)	131.0 (110.0, 146.0)	−6.303	<0.001
HCT (%)	55	53.2 (47.4, 59.8)	39.4 (33.2, 43.3)	−6.355	<0.001
MAP (mmHg)	55	95.0 (84.7, 102.0)	95.3 (84.7, 102.0)	0.310	0.757
HR (times/min)	55	107.0 (89.0, 125.0)	102.0 (89.0, 114.0)	−1.486	0.137
POP (mmol/L)	52	306.2 (297.7, 315.0)	298.0 (288.8, 309.1)	−3.652	<0.001
Lactic acid(mmol/L)	32	4.2 (3.1, 5.9)	1.7 (1.0, 2.7)	−4.576	<0.001
BUN (mmol/L)	53	6.9 (6.1, 8.5)	5.4 (3.6, 8.2)	−1.988	0.047
Serum creatinine (mg)	53	79.8 (69.6, 101.2)	65.6 (56.0, 75.3)	−3.214	0.001
Albumin (g/L)	55	33.4 (29.1, 38.4)	26.1 (24.3, 29.5)	−5.086	<0.001
ALT (U/L)	53	34.0 (24.2, 46.8)	28.1 (20.1, 34.7)	−3.606	<0.001
AST (U/L)	53	68.1 (56.2, 110.4)	34.2 (25.6, 49.1)	−5.747	<0.001
APTT (s)	41	32.6 (24.6, 59.1)	38.4 (30.8, 49.5)	−0.078	0.938

No.: number. HB: hemoglobin; HCT: hematocrit; MAP: mean arterial pressure; HR: heart rate; POP: plasma osmotic pressure; BUN: blood urea nitrogen; ALT: alanine aminotransferase; AST: aspartate aminotransferase; APTT: albumin and activated partial thromboplastin time. All data were compared by Wilcoxon signed rank test.

## Data Availability

The data that support the findings of this study are not openly available due to human data and are available from the corresponding author upon reasonable request.
